# Reply to Semin et al. Can Humans Discriminate Horse ‘Fear’ Chemosignals from Control Chemosignals? Comment on “Sabiniewicz et al. A Preliminary Investigation of Interspecific Chemosensory Communication of Emotions: Can Humans (*Homo sapiens*) Recognise Fear- and Non-Fear Body Odour from Horses (*Equus ferus caballus*). *Animals* 2021, *11*, 3499”

**DOI:** 10.3390/ani12121498

**Published:** 2022-06-09

**Authors:** Agnieszka Sabiniewicz, Michał Białek, Karolina Tarnowska, Robert Świątek, Małgorzata Dobrowolska, Piotr Sorokowski

**Affiliations:** 1 Institute of Psychology, University of Wrocław, 50-527 Wrocław, Poland; michal.bialek3@uwr.edu.pl (M.B.); k.a.c.tarnowska@gmail.com (K.T.); mrswiatek@wp.pl (R.Ś.); sorokowskipiotr@yahoo.co.uk (P.S.); 2 Department of Otorhinolaryngology, Smell and Taste Clinic TU Dresden, 01307 Dresden, Germany; 3 International Center for Interdisciplinary Research, Silesian University of Technology, 44-100 Gliwice, Poland; malgorzata.dobrowolska@polsl.pl

Whereas several recent studies demonstrated that some animal species are able to recognize human emotions based on information from body odor [1,2,3,4], our study [5] was the first to demonstrate that the ability to recognize emotions from body odor cues of other species might be reciprocal between animals and humans. In their critical comment [6], Semin and colleagues suggest that the study’s methodology should be changed. We would like to underline that the methodology of the first study in any field is always an initial attempt based on the researchers’ best knowledge and intentions and can be certainly improved in further studies.

We value the transparency of our data, which allow other researchers to discuss all of the typical methodological problems regarding studies in the field of odor-based interspecies communication. We are aware of the weaknesses of our study, as was pointed out in the manuscript itself. At the same time, even considering that the intensity of the odor samples partially modulated our findings, it should be noted that the intensity of odors is encoded in sweat [7]. To conclude, we invite other researchers to replicate our study.
